# Hemiclamshell Resection of a Ruptured Mediastinal Teratoma Exhibiting Pancreatic Differentiation

**DOI:** 10.1002/rcr2.70345

**Published:** 2025-09-18

**Authors:** Yuri Enomoto, Yoshio Nakano, Hiroyuki Fukuda, Yoko Yamamoto, Naoki Ikeda, Yumiko Yasuhara, Iwao Gohma

**Affiliations:** ^1^ Department of Respiratory Medicine Sakai City Medical Center Sakai Japan; ^2^ Department of Thoracic Surgery Sakai City Medical Center Sakai Japan; ^3^ Department of Pathology Sakai City Medical Center Sakai Japan

**Keywords:** hemiclamshell approach, islets of Langerhans, mediastinal teratoma, pancreas, surgical resection

## Abstract

Mediastinal teratomas demonstrating unidirectional pancreatic differentiation are exceedingly rare. We describe a 36‐year‐old woman with progressively worsening anterior chest pain. Computed tomography demonstrated a 5.9 cm multilocular cystic mass in the anterior mediastinum, accompanied by pleural and pericardial effusions. Given the lesion's size and presumed inflammatory reaction, we performed radical excision via a hemiclamshell incision. Histopathology revealed predominantly pancreatic acinar tissue with scattered non‐pancreatic epithelial elements, prompting reclassification as a mediastinal teratoma rather than ectopic pancreas. Focal epithelial disruption was regarded as the nidus of the surrounding inflammation. The patient's postoperative course was uneventful. This report underscores the need to include teratoma in the differential diagnosis of anterior mediastinal lesions initially interpreted as ectopic pancreas and illustrates the value of the hemiclamshell approach for achieving complete, atraumatic resection when extensive local adhesions are present.

## Graphical abstract

We report a rare anterior mediastinal teratoma exhibiting unidirectional pancreatic differentiation that ruptured microscopically, causing inflammation. Complete en bloc resection was achieved using a hemiclamshell approach without complications. Histology confirmed pancreatic acinar tissue, ducts, and islets of Langerhans.

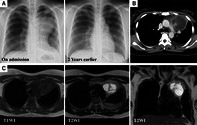

## 1 Introduction

Heterotopic pancreatic tissue is a developmental anomaly detected in approximately 2% of autopsies, predominantly within the gastrointestinal tract [1]. Its occurrence in the mediastinum—termed mediastinal ectopic pancreas (EP)—is extraordinarily rare. Some lesions previously labelled EP may in fact represent teratomas with predominant pancreatic differentiation [2]. We report such a tumour complicated by marked thoracic inflammation. Initial histological assessment revealed almost pure pancreatic tissue, leading to a provisional diagnosis of mediastinal EP. Comprehensive re‐evaluation disclosed minor non‐pancreatic epithelial components, confirming a teratoma exhibiting unidirectional differentiation toward pancreatic tissue. The mass was excised completely via a hemiclamshell incision without injury to adjacent structures.

## 2 Case Report

**FIGURE 1:**
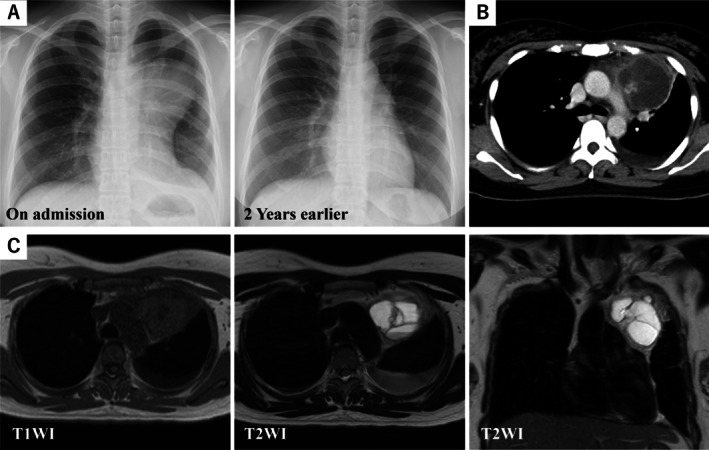
Radiological findings of the anterior mediastinal mass. (A) Chest radiograph revealing a new anterior mediastinal mass that was not present on imaging 2 years earlier. (B) Contrast‐enhanced CT showing a 5.9 × 6.1 × 6.8 cm anterior mediastinal mass, primarily composed of multilocular cystic components with minimal solid portions. Partial enhancement is seen along the internal septa. Left‐sided pleural effusion and pericardial fluid are also present. (C) MRI demonstrating low signal intensity on T1‐weighted images and high signal intensity on T2‐weighted images within the cysts. In contrast, the cyst walls appeared hypointense on both T1 and T2‐weighted images.

A 36‐year‐old Chinese woman presented with progressive stabbing pain in the anterior chest that intensified on inspiration and radiated to the left scapular region. Physical examination was unremarkable; however, laboratory tests showed leukocytosis (10,910/μL; reference range 3300–8600) and an elevated C‐reactive protein concentration (6.41 mg/dL; reference range 0.0–0.14). Electrocardiography demonstrated T‐wave inversions in leads V1–V3, whereas transthoracic echocardiography revealed preserved cardiac function. A chest radiograph revealed a new anterior mediastinal mass not seen on imaging two years earlier (Figure 1A). Contrast‐enhanced computed tomography (CT) demonstrated a 5.9 × 6.1 × 6.8 cm multilocular cystic lesion with scant solid components and subtle septal enhancement, together with left pleural and pericardial effusions (Figure 1B). Abdominal imaging was normal. We performed magnetic resonance imaging (MRI) to further characterise the mediastinal mass. MRI demonstrated low signal intensity on T1‐weighted images and high signal intensity on T2‐weighted images within the cysts. In contrast, the cyst walls appeared hypointense on both T1‐ and T2‐weighted images (Figure 1C). These findings suggested that the mediastinal mass was composed predominantly of fluid‐filled cysts and that the cyst walls likely lacked appreciable fat or proteinaceous components.

Given the tumour's size (> 5 cm) and the suspected severe intrathoracic inflammation, we elected to perform resection through a hemiclamshell approach to ensure optimal exposure. Intra‐operatively, yellow, turbid effusion was present within the pleural cavity. The mass adhered firmly to the chest wall, lung, pericardium, and phrenic nerve, yet no macroscopic invasion was evident. Careful dissection permitted en bloc removal without damage to neighbouring organs or nerves. Post‐operative recovery was uneventful, and diaphragmatic function remained intact.

**FIGURE 2:**
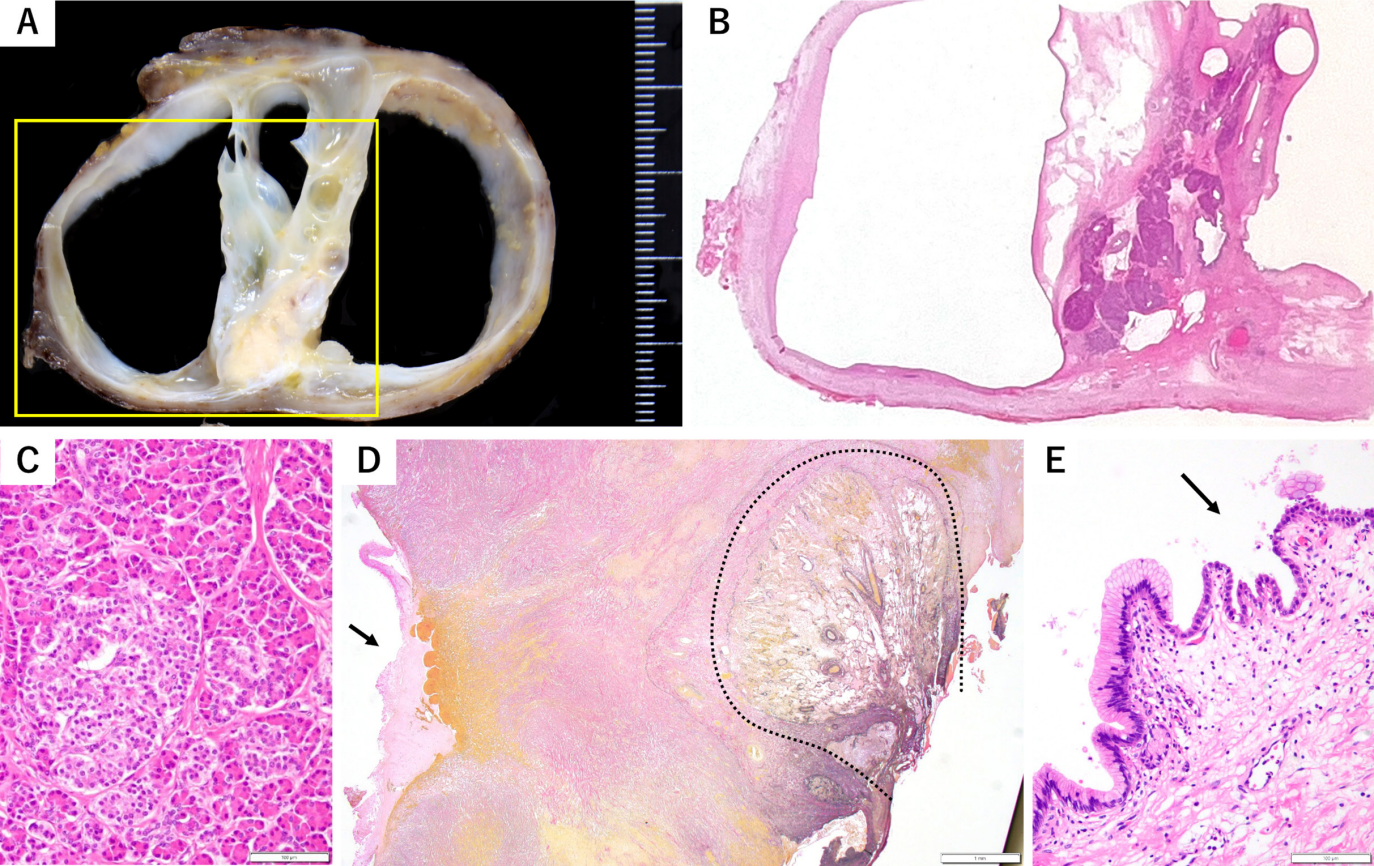
Histopathological findings of the resected mediastinal mass. (A) Cut surface of the resected mediastinal tumour. The specimen is a multilocular cystic tumour measuring approximately 6 cm in diameter, with a centrally situated thick yellow–whitish fibrous septum (yellow box). The cyst wall has a smooth surface. (B) Haematoxylin‐eosin (H&E)‐stained section of the area outlined by the yellow box in (A). The central septum is composed of organoid pancreatic tissue rich in well‐formed islets of Langerhans, and the adjacent cyst wall is lined by a single layer of tall columnar mucinous epithelium. (C) Higher‐magnification H&E‐stained view of the septum, showing pancreatic acini, ductal epithelium, and islets of Langerhans. Scale bar = 100 μm. (D) Elastic Van Gieson–stained section demonstrating focal epithelial disruption of the cyst wall, with accompanying haemorrhage and proliferating fibroblasts (arrow), and adhesion to the adjacent lung outlined by a dotted line. Scale bar = 1 mm. (E) H&E‐stained section of the cyst lining. Although most of the cyst lining consists of a single layer of columnar mucinous epithelium, a small portion of the cyst wall shows a transition to pseudostratified ciliated epithelium (arrow), forming contiguous regions of distinct epithelial types. Scale bar = 100 μm.

At gross examination, the resected specimen was a multilocular cystic tumour measuring approximately 6 cm in diameter, with a centrally situated thick yellow–whitish fibrous septum (Figure 2A). The cyst wall had a smooth surface and contained clear to brownish mucinous fluid. Microscopically, the central septum consisted of organoid pancreatic tissue rich in well‐formed islets of Langerhans, and the cyst wall was lined by a single layer of tall columnar mucinous epithelium (Figure 2B,C). The cyst wall showed focal epithelial disruption, with accompanying haemorrhage and proliferating fibroblasts, and was adherent to the adjacent lung (Figure 2D). Although most locules were lined by a single layer of columnar mucinous epithelium, thorough examination revealed a few areas in which the mucinous epithelium transitioned to pseudostratified ciliated epithelium, creating contiguous regions of distinct epithelial types (Figure 2E). In addition, small portions of the cyst walls displayed disorganised stratified squamous epithelium. These findings prompted revision of the diagnosis from EP to a mediastinal teratoma with predominant pancreatic differentiation. The microscopic breaches probably enabled inflammatory spread to the pleura, resulting in chest pain.

## 3 Discussion

We encountered an exceptional mediastinal teratoma with almost exclusive pancreatic differentiation that caused pleuritis and was successfully excised via a hemiclamshell incision without injury to adjacent structures. EP is defined as pancreatic tissue isolated from the native pancreas and devoid of ductal or vascular continuity [1, 3]. Mediastinal EP is extremely rare but has been described mostly in young Asian adults and usually in the anterior mediastinum [3]. It can enlarge considerably while remaining asymptomatic. Nonetheless, as Weichert et al. emphasised, at least a subset—and perhaps even the majority—of cases previously labelled as EP may in fact represent pancreatic‐predominant teratomas [2]. The pathogenesis of pancreatic differentiation within teratomas remains unresolved, but it may represent a neoplastic process rather than a developmental anomaly arising from the ventral foregut [2]. Accurate diagnosis requires not only identification of pancreatic tissue, but also careful recognition of teratomatous elements—such as skin appendages, cartilage, bone, or other derivatives of germ layers—via thorough histopathological sampling. Given the rarity of mediastinal teratomas with pancreatic differentiation and the many unresolved questions surrounding their pathogenesis, continued accumulation and analysis of additional cases will be essential moving forward.

Such tumours can compress or inflame adjacent structures, leading to pleural or pericardial effusions and chest pain [3]. In the majority of reported cases, bulky mediastinal masses have been resected via median sternotomy, although limited cases have been managed thoracoscopically [3, 4]. The hemiclamshell incision combines partial sternotomy with anterolateral thoracotomy, providing excellent exposure of the anterior mediastinum, pleural cavities, and lower neck, and is particularly advantageous for large tumours with dense adhesions or inflammation [5]. In our patient, this approach facilitated safe mobilisation of tissue adherent to the lung, pericardium, and phrenic nerve, enabling complete resection while preserving vital structures.

In conclusion, mediastinal teratomas with unidirectional pancreatic differentiation are extraordinarily rare, and some lesions previously regarded as EP may belong to this category. For bulky anterior mediastinal tumours accompanied by inflammation, the hemiclamshell incision affords reliable access for complete resection with minimal morbidity.

## Author Contributions

Yuri Enomoto managed the patient's initial treatment, conceived the case report, and drafted the manuscript. Yoshio Nakano provided direct supervision of the manuscript preparation. Iwao Gohma interpreted the data and revised the manuscript. Hiroyuki Fukuda, Yoko Yamamoto, and Naoki Ikeda performed the surgical resection of the mediastinal tumour and reviewed the manuscript. Yumiko Yasuhara interpreted the pathological findings. All authors approved the final version of the manuscript.

## Consent

The authors declare that written informed consent was obtained for the publication of this manuscript and accompanying images using the consent form provided by the Journal.

## Conflicts of Interest

The authors declare no conflicts of interest.

## Data Availability

Data sharing not applicable to this article as no datasets were generated or analysed during the current study.
